# The usefulness of Time-of-Flight MR angiography in detection of intraplaque hemorrhage in patients with acute ischemic stroke with symptomatic carotid stenosis

**DOI:** 10.1371/journal.pone.0229024

**Published:** 2020-02-13

**Authors:** Dong-Seok Gwak, Baik-Kyun Kim, Inyoung Chung, Moon-Ku Han

**Affiliations:** 1 Department of Neurology, Kyungpook National University Hospital, Daegu, Republic of Korea; 2 Department of Neurology, Chungbuk National University Hospital, Cheongju-si, Chungcheongbuk-do, Republic of Korea; 3 Department of Medicare System, Seoul National University Bundang Hospital, Seongnam-si, Gyeonggi-do, Republic of Korea; 4 Department of Neurology, Seoul National University Bundang Hospital, Seoul National University College of Medicine, Seongnam-si, Gyeonggi-do, Republic of Korea; Nagoya University, JAPAN

## Abstract

**Objective:**

Carotid intraplaque hemorrhage (IPH) is a well-known risk indicator of thromboembolism, but it is not easy to rapidly detect IPH in acute symptomatic carotid disease. The aim of this study was to assess the utility of time-of-flight (TOF) magnetic resonance angiography (MRA) in the detection of IPH and evaluate the degree of stenosis and stroke patterns in patients with acute symptomatic carotid disease.

**Methods:**

We retrospectively identified consecutive patients with acute symptomatic carotid disease who were admitted within 12 h after stroke onset. Fifty-nine patients underwent TOF MRA at admission and were categorized according to the presence or absence of intraplaque high signal intensity (HSI). The severity of carotid stenosis and diffusion-weighted magnetic resonance imaging lesion patterns were evaluated.

**Results:**

Intraplaque HSI was detected in 28.8% of the enrolled patients (17/59). Mild-to-moderate symptomatic carotid stenosis was more frequent in the intraplaque HSI-positive group (70.6%) than in the intraplaque HSI-negative group (42.8%) (p = 0.015). The patients with intraplaque HSI more frequently exhibited a disseminated small infarction pattern (76.5% in the intraplaque HSI-positive group, 47.6% in the -negative group), and did not exhibit a border-zone infarction pattern (0% in the positive group, 16.7% in the negative group).

**Conclusions:**

TOF MRA may be a useful noninvasive and rapid tool to detect IPH in patients with acute symptomatic carotid disease. IPH was common in those with a lower degree of carotid stenosis and manifested as a disseminated small infarction pattern. Intraplaque HSI on TOF MRA in acute symptomatic carotid disease may help to determine the mechanism of stroke and establish early treatment plans.

## Introduction

Extracranial carotid stenosis is a major risk factor for ischemic stroke [1]. This condition accounts for 20–30% of cases of ischemic stroke. However, approximately 70–80% of symptomatic patients with ≥50% carotid stenosis will not experience recurrent ipsilateral stroke within 5 years [2]. Thus, carotid disease should not be evaluated on the basis of the degree of stenosis alone. Not only the degree of stenosis, but also the vulnerability of the carotid plaque can cause a subsequent stroke event [3–5]. Vulnerable plaque is characterized by a lipid-rich necrotic core, thin fibrous cap, and remarkably, intraplaque hemorrhage (IPH) [6, 7]. IPH induces growth of the lipid-rich necrotic core and destabilizes atherosclerotic plaques [8]. Further, carotid IPH is highly associated with recurrent ischemic symptoms [9].

Carotid IPH can be measured by various methods such as high-resolution multi-contrast magnetic resonance imaging (MRI), time-of-flight magnetic resonance angiography (TOF MRA), and duplex sonography. Among these methods, TOF MRA evaluates carotid IPH rapidly without any additional MRI sequence. IPH appears as high signal intensity (HSI) around the carotid artery with this technique and can thus be detected easily. TOF MRA also detects IPH noninvasively and accurately compared to high-resolution multi-contrast MRI [10]. Because of these advantages of the TOF MRA sequence, it is a suitable method for evaluating the acute stage of ischemic stroke. This method can help to determine the mechanism of stroke by detecting relevant carotid artery stenosis and can aid the establishment of early treatment plans.

Carotid IPH is considered a risk factor for ischemic stroke, but its influence on the development of specific stroke patterns and association with the degree of carotid stenosis are unclear. Using TOF-MRA, we aimed to investigate the degree of stenosis and stroke patterns according to the presence of IPH in patients with acute symptomatic carotid artery disease who were admitted within 12 h of stroke onset.

## Methods

### Study populations

We retrospectively analyzed the data of 4,907 patients with ischemic stroke who were admitted to the Seoul National University Bundang Hospital between April 2006 and July 2012, from the prospective institutional stroke registry. We included patients who met the following criteria: (1) patients who visited our hospital within 12 h of symptom onset (n = 2332), (2) had symptomatic carotid artery disease (n = 110), and (3) underwent brain MRI with a TOF MRA sequence (n = 78). Symptomatic carotid disease is defined as the presence of carotid plaque that causes focal neurologic symptoms such as ipsilateral transient ischemic attacks or ipsilateral hemispheric stroke. Patients with cardioembolic etiologies such as atrial fibrillation were excluded, because the possible etiologies of stroke of these patients were two or more. We also excluded patients who underwent endovascular treatment prior to diffusion-weighted MRI, which could influence the infarct patterns. Finally, 59 patients were eligible for the analyses. Baseline demographic and clinical information including age, sex, body mass index, hypertension, diabetes, dyslipidemia, atrial fibrillation, smoking history, previous stroke history, previous medication history, including antiplatelet agents and statins, statin use on admission, and baseline National Institutes of Health Stroke Scale scores were gathered from the stroke registry. Laboratory data such as fasting blood glucose, glycated hemoglobin, total cholesterol, triglyceride, high density lipoprotein, and low-density lipoprotein were also collected. Patients who were treated with intravenous or intra-arterial thrombolysis were checked.

### Analysis of carotid disease

**Fig 1:**
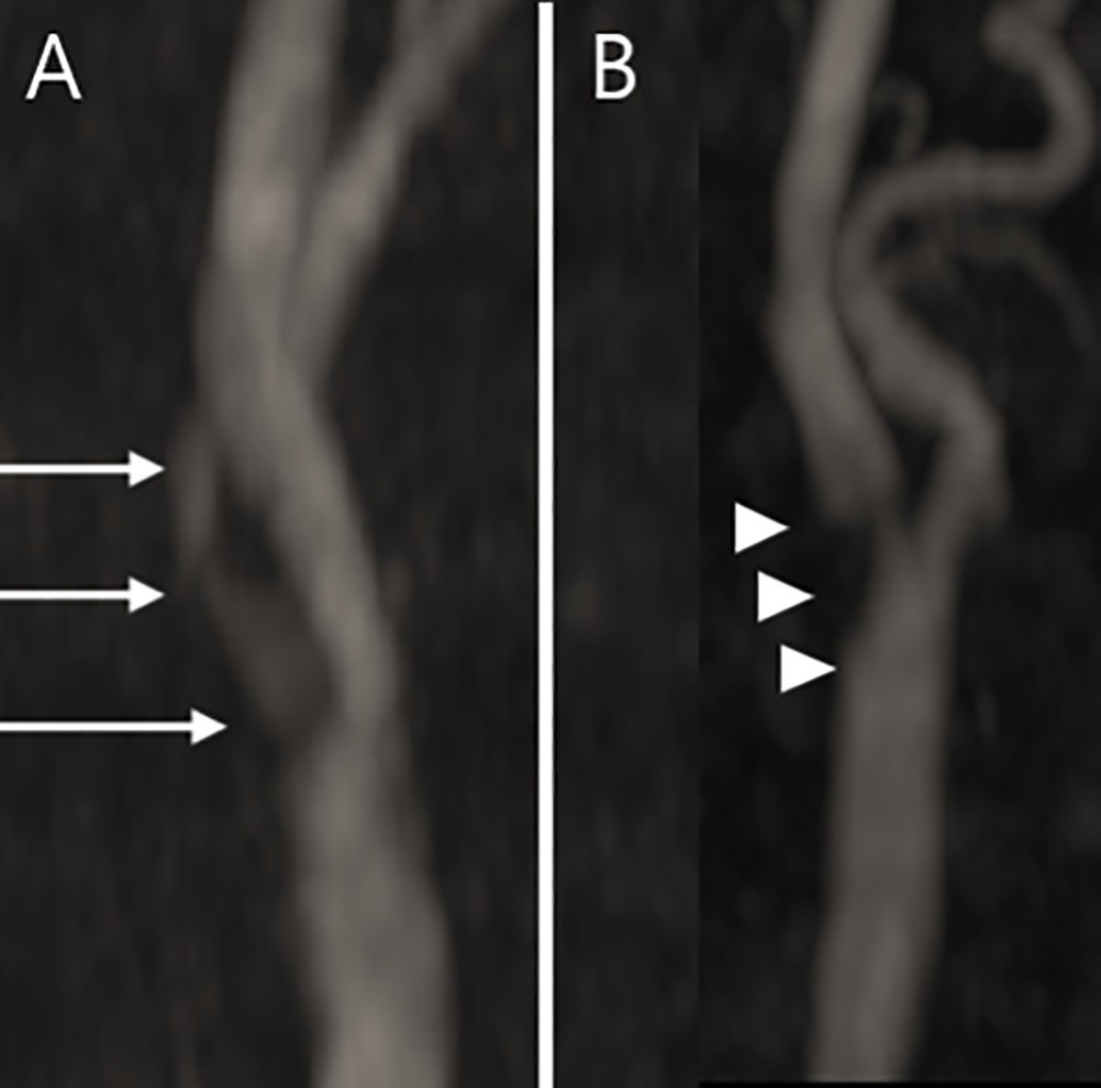
Time-of-flight magnetic resonance angiography images of the carotid arteries. A. Intraplaque hemorrhage (IPH) appeared as a high signal intensity halo (halo sign) around the proximal portion of the internal carotid artery without connection to the lumen (white arrows). B. Carotid stenosis without a halo sign was classified as the IPH negative group (white arrowheads).

MRI was performed using either 1.5-T systems or 3-T systems (Intera Achieva, Philips Healthcare). Neck TOF MRA was generated in the axial plane using the following parameters to obtain carotid artery images: repetition time (TR)/echo time (TE) = 23-24/4-5 ms, flip angle = 16°, field of view (FOV) = 75 × 150 mm, matrix = 320 × 164, slice thickness = 1.8 mm (0.9 mm overlap with adjacent section), and section slices = 120. A 20-mm-thick presaturation band, 10 mm above the imaging stack, was applied to saturate the venous blood on neck TOF MRA. Carotid IPH and the degree of stenosis of the carotid artery were measured by a neurologist. The patients who exhibited a HSI halo around the carotid artery on the TOF MRA image were classified as the intraplaque HSI-positive group, whereas the patients who did not were classified as the intraplaque HSI-negative group (Fig 1) [10]. Carotid stenosis on TOF MRA was evaluated according to the North American symptomatic carotid endarterectomy trial criteria [2]. The severity of carotid stenosis was divided into four categories: mild (<50% stenosis), moderate (50–69% stenosis), severe (70–99% stenosis), and occlusion (100% stenosis).

### Analysis of infarct patterns

**Fig 2:**
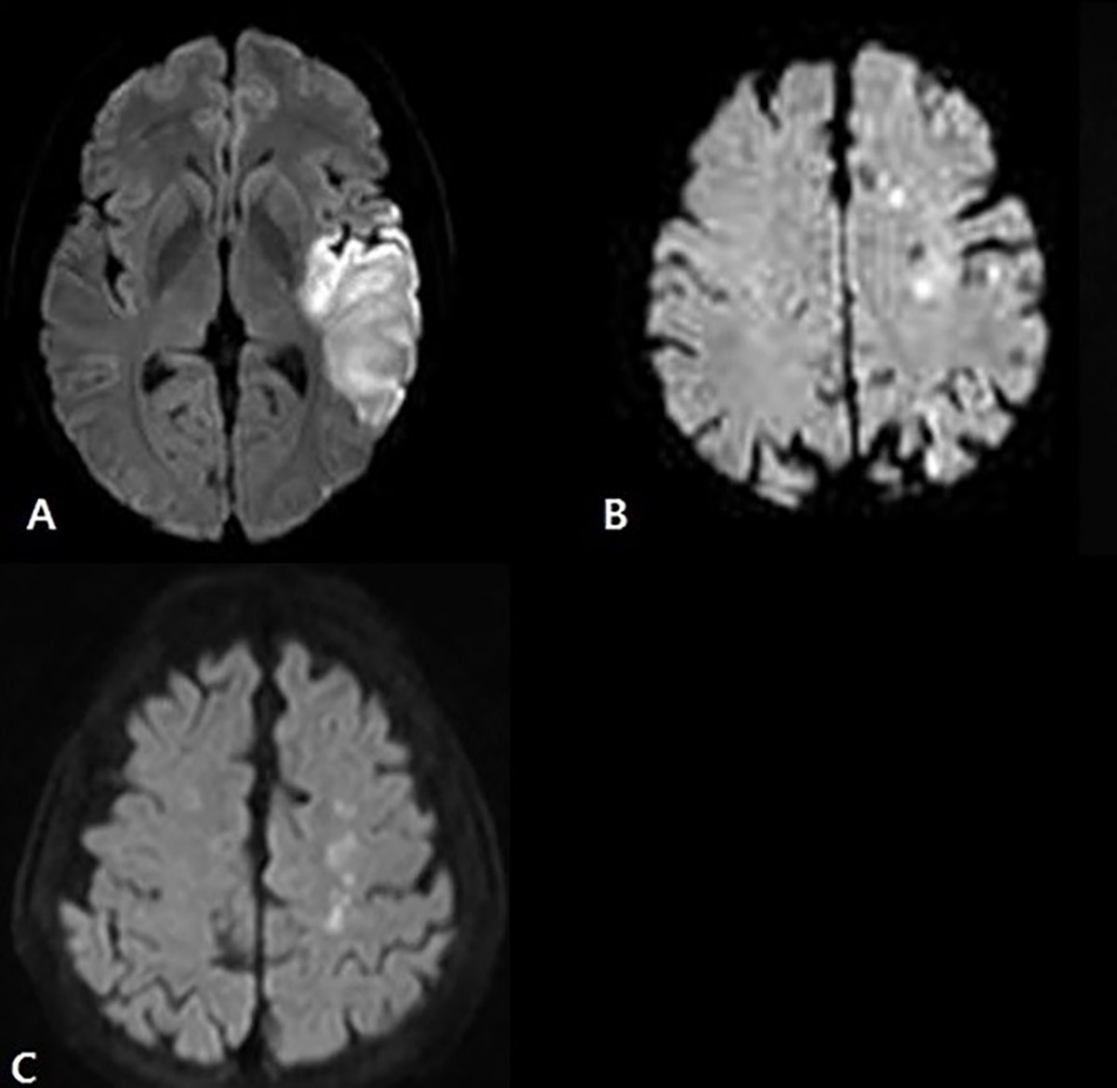
Three infarct patterns on diffusion-weighted magnetic resonance images. A. Large territorial lesion. B. Disseminated small lesions. C. Border zone infarction.

We reviewed the patterns of diffusion-weighted imaging (DWI) lesions. Infarct lesions were classified as one of three patterns based on the lesion size, location, and distribution: a large territorial lesion, disseminated small lesions, and border zone infarction (Fig 2). A large territorial lesion is defined as wedge-shaped lesion involving the cerebral cortex and subcortex. Disseminated small lesions are randomly scattered in the distal middle cerebral artery (MCA) or anterior cerebral artery (ACA) territory. Border zone infarction represents lesions mainly located between the territories of the two major cerebral arteries. This infarct pattern consists of anterior cortical (between the MCA and ACA), posterior cortical (between the MCA and posterior cerebral artery), or the internal border zone (between the superficial and deep arterial systems).

### Statistical analysis

Data are presented as the number (%) or mean ± standard deviation. To compare the baseline characteristics between the intraplaque HSI-positive and -negative groups, the chi-square test or Fisher’s exact test were used for parametric variables, Mann-Whitney U test for non-parametric variables, and student’s t-test for continuous variables. The chi-square test was used to determine the relationship between the presence of IPH and the degree of carotid stenosis and the infarct patterns. Potential confounding variables were adjusted in a logistic regression model to eliminate the effects of confounding factors. A two-tailed p-value <0.05 was considered statistically significant, and all statistical analyses were performed with SPSS for Windows, version 18.0.

### Ethics statement

This study was approved by regional institutional review boards (IRB) of Seoul National University Bundang Hospital, Republic of Korea (IRB No. B-1812/510-103) and written informed consent was waived from all study participants owing to the retrospective design of this study. All patients information were de-identified and anonymized prior to analysis.

## Results

### Study populations

A total of 59 patients with symptomatic carotid disease were enrolled in this study. There were 45 men (76.3%) and 14 women (23.7%). The mean age was 70.9 ±11.4 years. The interval time between the onset of stroke and arrival at the hospital was 4.10 ± 3.38 h.

On TOF MRA images, 17 patients (28.8%) exhibited a HSI halo sign at the proximal part of the internal carotid artery and were classified as the intraplaque HSI-positive group (Fig 1). The remaining 42 patients (71.2%) who did not exhibit the HSI halo sign at the proximal part of the internal carotid artery were classified as the intraplaque HSI-negative group. The baseline characteristics of both groups are compared in Table 1. History of stroke was less prevalent in the intraplaque HSI-positive group (5.9%) than the -negative group (31.0%) (p = 0.048). Other characteristics including cerebrovascular risk factors, initial laboratory data, the proportion of patients who were treated with intravenous or intra-arterial thrombolysis, and history of medication use did not significantly differ between the two groups.

**Table 1:** Baseline demographics and clinical characteristics.

Variable	Total (n = 59)	IPH(+) (n = 17)	IPH(-) (n = 42)	P-value
**Male,**	45 (76.3%)	14 (82.4%)	31 (73.8%)	0.737
**Mean age (years±SD)**	70.9±11.4	73.9±12.7	69.6±10.7	0.197
**BMI (kg/m^2^±SD)**	23.0±3.1	23.1±3.3	22.9±3.0	0.770
**Interval from onset to presentation (h±SD)**	4.10±3.38	3.73±3.50	4.25±3.37	0.498
**Within 3 h,**	28 (47.5%)	9 (52.9%)	19 (45.2%)	0.931
**3–6 h,**	14 (23.7%)	4 (23.5%)	10 (23.8%)	
**6–12 h,**	17 (28.8%)	4 (23.5%)	13 (31.0%)	
**Initial NIHSS score (mean±SD)**	4.29± 4.33	4.00±4.39	4.40±4.36	0.794
**Concomitant disease,**				
**History of stroke**	14 (23.7%)	1 (5.9%)	13 (31.0%)	0.048
**Hypertension**	47 (79.7%)	16 (94.1%)	31 (73.8%)	0.150
**Diabetes mellitus**	18 (30.5%)	7 (41.2%)	11 (26.2%)	0.350
**Hyperlipidemia**	18 (30.5%)	5 (29.4%)	13 (31.0%)	1.000
**Smoking**	26 (44.1%)	6 (35.3%)	20 (47.6%)	0.563
**Atrial fibrillation**	0 (0%)	0 (0%)	0 (0%)	-
**Thrombolysis,**	12 (20.3%)	3 (17.6%)	9 (21.4%)	1.000
**IV**	6 (10.2%)	2 (11.7%)	4 (9.5%)	
**IA**	4 (6.8%)	0 (0%)	4 (9.5%)	
**IV+IA**	2 (3.4%)	1 (5.9%)	1 (2.4%)	
**Laboratory data**				
**FBS (mg/dl±SD)**	111.4±39.6	117.5±37.3	108.9±40.6	0.162
**HbA1c (%±SD)**	6.4±1.7	6.95±2.64	6.18±1.10	0.435
**TC (mg/dl±SD)**	180.4±51.0	171.7±42.3	184.0±54.1	0.405
**TG (mg/dl±SD)**	121.3±65.6	111.2±64.1	125.4±66.6	0.186
**HDL (mg/dl±SD)**	44.3±13.2	45.6±13.3	43.8±13.2	0.446
**LDL (mg/dl±SD)**	111.8±44.7	103.8±33.6	115.1±48.5	0.386
**Previous statin use, n (%)**	12 (20.3%)	3 (17.6%)	9 (21.4%)	1.000
**Antiplatelet drug use (initial), n (%)**				0.396
**Aspirin**	12 (20.3%)	3 (17.6%)	9 (21.4%)	
**Clopidogrel**	0 (0%)	0 (0%)	0 (0%)	
**Aspirin + Clopidogrel**	46 (78.0%)	13 (76.5%)	33 (78.6%)	
**Others**	1 (1.7%)	1 (5.9%)	0 (0%)	
**Statin use on admission**	55 (93.2%)	14 (82.4%)	41 (97.6%)	0.068
**Intervention within 1 year**	31 (52.5%)	8 (47.1%)	23 (54.8%)	0.774

Values are presented as the mean±standard deviation (SD) for continuous variables or as the n (%) of participants for categorical variables.

P-values were calculated by Pearson’s chi-square test, Fisher’s exact test, the Whitney Mann U test, and the Student’s t-test according to the variable’s characteristics

Abbreviation: IPH, intraplaque hemorrhage; BMI, body mass index; NIHSS, National Institutes of Health Stroke Scale; IV, intravenous; IA, intra-arterial; FBS, fasting blood glucose; HbA1c, glycated hemoglobin; TC, total cholesterol; TG, triglyceride; HDL, high density lipoprotein; LDL, low density lipoprotein

### Analysis of carotid disease

Table 2 presents the degree of carotid stenosis in the intraplaque HSI-positive and -negative groups. The intraplaque HSI-positive group had a lower degree of carotid stenosis than the -negative group (p = 0.015). The prevalence of mild to moderate symptomatic carotid stenosis was 70.6% (12/17) in the intraplaque HSI-positive group and 42.8% (18/42) in the negative group. However, the prevalence of mild to moderate symptomatic carotid disease was not significantly different between the two groups when analyzed without occlusion cases (70.6% (12/17) in the positive and 64.3% (18/28) in the negative group.)

**Table 2:** Degree of stenosis of the carotid artery in the high signal intensity-positive and -negative groups.

Variable	Total (n = 59)	HSI(+) (n = 17)	HSI(-) (n = 42)	P-value
**Severity of carotid stenosis, n (%)**				0.015^a^
**Mild**	14 (23.7%)	4 (23.5%)	10 (23.8%)	
**Moderate**	16 (27.1%)	8 (47.1%)	8 (19.0%)	
**Severe**	15 (25.4%)	5 (29.4%)	10 (23.8%)	
**Occlusion**	14 (23.7%)	0 (0%)	14 (33.3%)	

HSI, high signal intensity. Values are presented as n (%).

^a^p-values were calculated by Fisher’s exact test.

### Analysis of infarct patterns

Table 3 describes the different infarct patterns on DWI between the intraplaque HSI-positive and -negative groups. The presence of disseminated small infarct patterns (odds ratio [OR]: 3.58, 95% confidence interval [CI]: 1.00–12.78; p = 0.050) was more frequent in the intraplaque HSI-positive group than the -negative group. Border-zone infarction patterns were less frequent in the intraplaque HSI-positive group than the -negative group (0% vs. 16.7%). The prevalence of large territorial lesion patterns did not differ between the two groups (OR: 0.55, CI: 0.15–2.00; p = 0.368).

**Table 3:** Infarct patterns in the high signal intensity-positive and -negative groups.

Infarct patterns	HSI(+) (n = 17)	HSI(-) (n = 42)	Unadjusted OR (95% CI)	P-value^b^	Adjusted OR^a^ (95% CI)	P-value^b^
**Large territorial**	4 (23.5%)	15 (35.7%)	0.55 (0.15–2.00)	0.368	1.60 (0.34–7.58)	0.554
**Small, disseminated**	13 (76.5%)	20 (47.6%)	3.58 (1.00–12.78)	0.050	1.72 (0.41–7.21)	0.458
**Border zone**	0 (0%)	7 (16.7%)	NA	NA-	NA	NA

The odds ratio (OR) for the high signal intensity (HSI)-positive group over the high signal intensity-negative group.

^a^The degree of stenosis was adjusted for the multivariable logistic regression analysis.

^b^P-values were calculated by Pearson’s chi-square test or Fisher’s exact test according to the variable’s characteristics

CI, confidence interval

NA denotes for not available because of the small number of events, resulting in unreliable estimates.

## Discussion

In this study, we used a TOF MRA sequence to evaluate carotid IPH. As mentioned above, this method has several advantages in evaluating the hyperacute stage of patients with stroke in that it can detect IPH rapidly, and noninvasively. Moreover, since TOF MRA is commonly used technique for carotid evaluation without additional high-resolution surface coils, it can be easily applied to clinical practice. If carotid IPH is detected early using TOF MRA in patients with a mild to moderate degree of symptomatic carotid stenosis, it is possible to determine the etiology of stroke (artery to artery thromboembolism from the carotid artery) and establish an early treatment plan for atherosclerosis with this method.

In our study, the prevalence of mild to moderate symptomatic carotid stenosis tend to be higher in IPH positive than negative group, although the difference was not significant when analyzing except occlusion cases. One previous study showed that the overall carotid stenosis evaluated by TOF MRA was similar between the IPH positive and negative groups (41.0% vs 44.6%, p = 0.649) [11]. This study enrolled patients with both symptomatic and asymptomatic carotid diseases. Since our study only enrolled the patients with symptomatic carotid diseases and IPH may contribute to the vulnerability of the plaque, those with a lower degree of carotid stenosis tended to be more prevalent in the IPH positive groups.

Although previous studies had reported the relationship of carotid IPH with regional acute cerebral ischemic events, neither of these studies demonstrated the pattern of infarction associated with IPH [12, 13]. We found that carotid IPH may influence the infarct patterns of patients with symptomatic carotid disease. The patients with carotid IPH were more likely to exhibit disseminated small infarct patterns. Because multiple small disseminated lesions are caused by the fragmentation of thrombi or multiple emboli [14], we could deduce that carotid plaque in patients with IPH have a tendency to produce more microthrombi than other characteristics of carotid plaque. The patients with carotid IPH did not exhibit border-zone infarction patterns, but caution should be exerted in interpreting the result because the intraplaque HSI-positive group had no carotid occlusion cases and the degree of carotid stenosis can be a potential confounder [15, 16].

Our study has some limitations. This was a retrospective observational study that was susceptible to bias in the data collection and analysis. In addition, this study design cannot confirm causal relationships among variables, although it is possible to identify the associations among them. Confounding variables that can adversely affect the correlation between the independent variable and dependent variable may exist. For example, because both carotid IPH and ulcerative plaque increase the risk of plaque rupture, the etiology of stroke in patients with symptomatic carotid IPH and ulcerative plaque is undetermined. We did not adjust such variables. Further, if the degree of carotid stenosis is mild or the IPH did not develop recently, the lack of a halo sign could be a false negative [17].

In summary, our study demonstrated that the TOF MRA sequence plays a significant role in the detection of IPH in patients with acute symptomatic carotid disease. IPH was common in those with a lower degree of carotid stenosis. We also observed that the intraplaque HSI-positive and -negative groups exhibited different infarct patterns. The small disseminated infarction pattern was more frequent in the intraplaque HSI-positive than the -negative group, which suggested the IPH may produce more microthrombi than other characteristics of the plaque. However, further well-designed clinical studies are needed to evaluate the relationship between the presence of IPH and the degree of stenosis and stroke patterns in patients with early symptomatic carotid disease.

## Supporting information

S1 Dataset The data set of this study. The data set include the data and coding sheet of all study subjects.
(XLSX)
